# Supplementary material for: Are carbon emissions trading and green financial instruments synergistic? -Comprehensive quantitative research based on content analysis
Source: PLoS One. 2024 Mar 7;19(3):e0298601. doi: 10.1371/journal.pone.0298601 (PMC10919601; doi:10.1371/journal.pone.0298601)
Supplement: S1 Data — (ZIP) [file pone.0298601.s001.zip › Data/地级市Z_F工作报告.zip!/╜¡╬≈/╨┬╙α╩╨/└┤╫╘╣1⁄2╓┌║┼í╛┬φ┐╦╩2╛▌═°í┐_2017.docx]

各位代表：

　　现在，我代表市人民政府向大会报告政府工作，请予审议，并请各位市政协委员和列席会议的同志提出意见。

　　一、2016年工作回顾

　　过去的一年，面对错综复杂的宏观环境、经济下行的外部压力和各种风险挑战的叠加冲击，在省委、省政府和市委的正确领导下，全市上下紧紧围绕打赢翻身仗总目标，团结拼搏，奋发进取，实现了“十三五”良好开局。预计全年实现生产总值1000亿元，增长8.6%；财政总收入137.2亿元，增长3.3%（同口径增长11%）；规模以上工业增加值321亿元，增长9%，主营业务收入1640亿元，增长5%；固定资产投资921.3亿元，增长12%；社会消费品零售总额237.7亿元，增长11.2%；出口总额12.9亿美元，增长2.5%；实际利用外资4亿美元，增长9%，省外2000万元以上项目资金423.2亿元，增长12%；城镇居民人均可支配收入32375元，增长8.5%，农村居民人均可支配收入15245元，增长9%；居民消费价格涨幅控制在1.5%。

　　(一)求新思变，在着力培育新的经济增长点中产业结构不断优化。工业经济提质发展。坚持工业主导不动摇，以供给侧结构性改革为突破口，从生产端发力，大力实施项目带动战略，集中培育集聚性、引领性和带动性强的龙头企业，逐步形成了钢铁、新能源、光电信息、装备制造等四大主导产业，主营业务收入分别增长12%、42%、65%、30%。坚持产业集群引领转型升级，大力实施锂电、手机触控、LED、装备制造等产业集群三年倍增计划，深入开展产业集群公共服务平台建设三年行动计划，以战略性新兴产业为核心的产业集群效应初步显现。坚持加大工业运行调度，强化工业要素保障，工业用电量增长5.5%，铁路货运量增长11.6%，规模以上工业实现利税增长18.0%、利润增长32.9%，工业经济效益综合指数达296.8%、提高9.8个百分点，工业经济效益显著提升。坚持以工业园区为主战场，着力完善基础设施，创新运行体制机制，提升公共服务水平，新建标准厂房35万平方米，园区规模以上工业增加值占比达70%。坚持以企业需求为导向，扎实开展精准帮扶，设立产业引导专项资金3亿元，发放信贷通贷款总额9.1亿元，市级还贷周转金规模达1.48亿元，帮助企业倒贷402笔、61亿元；积极开展降成本优环境专项行动，对照“省80条”政策措施梳理出台市实施细则83条，帮助企业降低成本30.6亿元。赛维公司破产重整取得重大突破。成功引进世界五百强企业美国雅保收购江锂新材料。新增规模以上工业企业58户。第三产业提速发展。实现增加值390亿元、增长8.5%，新增规模以上服务业企业28户。全市金融机构人民币存、贷款余额分别增长6.3%和7.5%，各项融资余额1521.2亿元、增长8.6%。新增百乐米业、增鑫牧业等2家企业挂牌“新三板”。新引进分宜九银村镇银行和兴业银行渝水小微支行，实现村镇银行县域全覆盖和股份制商业银行引进零突破。新余苇禾保理公司成功开业，实现融资担保模式新突破。电子商务实现交易额260亿元，增长62%。旅游业接待游客增长21.2%，旅游收入增长20.7%。成功列入国家第二批全域旅游示范区创建名单，中国洞都成功创建国家4A级旅游景区，凯光亚热带植物园、昌坊旅游度假村列为省级乡村旅游点。文化产业增加值增长29%，占生产总值比重提高到3.62%。国际麻纺博览会、南方农业（工程）机械展销会影响力日益提升。居家养老、家政服务、医疗保健等服务业态蓬勃发展。现代农业集约发展。粮食生产稳定，农牧渔业全面增产。52家省级以上农业产业化龙头企业实现销售收入129.4亿元、增长9.8%，新增省级农业产业化龙头企业3家、农民合作社示范社7家、示范性家庭农场30家。特色农业态势良好，新余蜜桔、优质葡萄、麒麟西瓜等产销两旺。罗坊沼气二期工程竣工，新增集中供气农户1500户，成为全省发展生态循环农业的样板。科技创新助推发展。科技进步对经济增长贡献率达56%。共获省科学技术进步奖5项、省技术发明奖1项。新增博士后创新实践基地2家、省级工程技术研究中心2家，获批国家和省级众创空间各1家。新增国家高新技术企业15家。申报省级以上科技项目46项，开发省级重点新产品41项。专利申请量1620件、授权量1203件，分别增长11%和35.5%。新增国家科技创新创业人才1人、省主要学科学术和技术带头人2人、省“百千万人才工程”人选2人。

　　(二)固本强基，在着力推进项目建设中发展后劲持续增强。招商引资成效明显。强化领导招商，突出产业招商，坚持以商招商，共引进5000万元以上项目129个、金额332.4亿元，其中赣锋电池、沐阳科技等投资10亿元以上项目8个，新签约项目开工率达88.8%。重点项目进展顺利。安排市重点项目187个，完成投资150亿元，顺利实现项目个数和投资总额“两个翻番”目标。着力创新项目推进机制，强化重点项目分级管理，成立工业、城建等七个项目工作小组，实行分级负责、分口把关、密集调度、协同推进，卓照光电LED系列灯具、新联泰车轮钢圈一体化等项目进展顺利，木林森LED照明产品、佳沃动力电池等项目建成投运。列入省重点建设项目7个，其中蒙华铁路（新余段）、国电投分宜电厂等重大项目进展顺利。新增光伏发电并网容量331.9兆瓦。政策资金争取得力。共争取上级资金74.4亿元。“城市矿产”示范基地通过国家终期验收，获清算补助资金9200万元；国家资源枯竭城市转型试点绩效考核通过省级论证，获中央转移支付资金2.7亿元。仙女湖湖泊环境整治三年评估通过国家验收，获财政奖励资金2.73亿元。孔目江流域洪水防控和环境治理项目1.5亿美元亚行贷款获批。获批政府性债务置换资金39.2亿元，政府债务还本付息压力降低。

　　(三)协调联动，在着力推进城乡统筹中居住环境明显改善。规划体系不断健全。积极参与《新宜萍城镇群战略规划》《环鄱阳湖生态城市群规划》等上位规划编制，完成了消防、电网、高铁新区地下综合管廊等专项规划编制，启动了海绵城市总体规划、孔目江新城控制性详规等编制工作。城市功能不断完善。全年安排城建项目39个，完成投资12.5亿元。新欣大道、天工大道、环城南路、五一路等改造工程全面完成，百乐路、塘下路、清新路、伴山路等建成通车，珊娜大桥开工建设。北湖公园东出入口及配套工程、北湖公园至仰天岗纪念碑广场城市绿轴、孔目江湿地公园三期等项目扎实推进，廖家江改道工程、贯早江水渠改造景观工程、暨阳人民广场、公园夜景改造提升工程等全面完成。12处背街小巷、17个老旧小区、7个公产房片区、4个城中村基础设施改造全面完成。吉泰苑保障房全面建成，新钢路东棚户区、中洲棚户区、新纺棚户区改造项目稳步推进。中心城区雨污分流改造一期项目基本建成，完成排水管网隐患排查50.6公里。改造公交站台42座，建设公交智能电子站牌80座，新增纯电动公交车26辆。公共自行车系统建成投运。城区环卫、园林绿化、市政管护等工作扎实开展，城市管理水平持续提升。城乡一体融合发展。余新公路建成通车，欧里至东岳庙公路及人和至洞村连接线工程基本建成，国省道一级公路绕城项目前期工作全面启动。城市电网中低压改造、农村电网升级改造扎实推进，城乡供电保障水平不断提高。中心镇建设有序推进，集镇综合服务功能进一步提升。首批19个中心示范村建设任务全面完成。农村生活垃圾专项治理扎实推进，实现治理设施、保洁队伍和常态化考核管理全覆盖。完成土地开发整理4855亩，新增耕地2728亩。生态环境不断改善。超额完成省下达的单位GDP能耗下降任务。大气污染防治深入推进，新钢烧结机烟气脱硫旁路取消工程、城西污水处理厂等重点减排项目建设全面完成，城区空气质量优良率达93.1%。生活垃圾焚烧发电厂运行良好，实现新余全境生活垃圾处理无害化、减量化和资源化。“河长制”工作扎实推进，袁河新余段、孔目江下游水质不达标河段、袁惠渠等水环境综合整治成效明显，仙女湖水体污染突发事件妥善处置。“五山”整治和生态修复成效显著。新增造林4.9万亩，荣获全国绿化模范城市称号。

　　(四)以民为先，在着力保障和改善民生中社会发展更加和谐。40件民生实事全面完成。就业创业成效明显。出台了大众创业万众创新系列配套政策，新增城镇就业2.3万人，转移农村劳动力1.5万人，城镇登记失业率控制在3.3%。新发放创业担保贷款3.6亿元，扶持2466人创业，带动1.1万人就业。社会保障更加有力。机关事业单位养老保险制度改革稳步推进。城乡医疗保险制度加快整合，新农合年人均筹资标准提高到540元、参合率达99.5%，城镇居民基本医疗保险年人均筹资标准提高到540元，城镇职工大病医疗保险最高支付限额提高到30万元。城市低保、农村低保保障标准分别提高6.7%和12.5%，农村“五保”集中供养补助标准提高19.7%，城镇“三无”特困群众供养标准月人均提高到600元。市养老福利中心二期主体工程竣工；农村“颐养之家”建设全面推进，已在48个村委建立“颐养之家”88个。发放廉租住房租赁补贴5660户、732.2万元。大力推进精准扶贫、精准脱贫，年度1万人脱贫目标超额完成；建成光伏扶贫项目8.4兆瓦。社会事业蓬勃发展。分宜县、渝水区、仙女湖区顺利通过国家义务教育基本均衡发展督导评估认定，城区学校“片区+盟校”办学模式等改革成为全省义务教育先进典型案例。高考成绩斐然，本科上线比例达48.3%，增长15.1个百分点。职业教育内涵提升，现代学徒制改革试点扎实推进。少儿艺术、幼儿基本体操蝉联全国冠军，校园足球水平走在全省前列。城市公立医院综合改革成效显著，分级诊疗制度全面推行。成功创建国家公共文化服务体系示范区。全面两孩政策落实到位。全民健身运动广泛开展，成功举办第八届市运会、环仙女湖国际马拉松等赛事。社会治理不断加强。全国文明城市创建有序推进，“新余有爱”志愿活动蓬勃开展。蝉联全国双拥模范城“七连冠”和全省“八连冠”。法治新余建设深入推进，“七五”普法全面启动。畅通信访渠道，群众合理诉求得到有效解决。平安新余建设扎实推进，在全省率先启动新警综平台建设，应急预警机制进一步完善，国家安全人民防线进一步加强，没有发生较大及以上安全事故，没有发生重大群体性事件，没有发生重特大刑事案件，全市社会和谐稳定。统计、审计、海关、检验检疫、民族宗教、外事侨务、涉台事务、档案、地方志、机关事务、防灾减灾、妇女儿童、青少年、老龄、科普、气象、人防、邮政通讯、烟草、盐业、海事、民兵预备役、社科研究、红十字会人道援助和慈善、残疾人事业等工作取得新成绩。

　　(五)先行先试，在着力深化改革创新中市场活力加速释放。32项重点改革项目年度任务全面完成。行政管理体制改革扎实推进。顺利承接地方立法权，政府立法工作全面启动。深化绩效考核制度改革，实现绩效考核全程化、信息化和全覆盖。推进机构编制管理改革，部门内设机构进一步整合。“三单一网”改革全面推进，便民服务事项办事指南编制完成。在全省率先开展“一窗式”网上审批服务改革，审批效能大幅提升。积极推进“五证合一、一照一码”商事制度改革，新登记各类市场主体5718户，新增注册资本198.3亿元。经济体制改革稳步推进。农村土地确权登记颁证工作稳妥推进。供销合作社综合改革启动。超额完成省下达化解煤炭过剩产能任务。商品房去化周期缩短27.5%，城市棚户区改造货币化安置率达73%。持续推进预算绩效管理改革和全口径预算制度改革，预算支出定额标准体系逐步健全，市本级专项资金压缩34.2%。经营性国有资产集中统一监管改革全面完成，国有资产保值增值率达102%。社会管理体制改革深入推进。社区数字化综合服务管理平台实现城区社区全覆盖。新钢社区管理等社会职能承接工作持续深化。社会信用体系基本建成，信用新余正式上线运行。智慧城市建设成效显著，在全省率先进入“全光网城市”，国家智慧城市发展研究中心江西分中心在新余挂牌，智慧时空信息云平台列入国家试点，智慧校车成为全省样板。

　　各位代表！过去的一年，我们坚持依法行政，进一步完善了政府议事规则、行政决策、考核问责等方面的工作机制，有力地推动了行政权力在法治轨道上运行。我们坚持问题导向，深入开展“两学一做”学习教育，坚决落实中央八项规定精神，开展了“红包”问题、“违插”问题等专项治理，集中解决了一批人民群众反映强烈的热点难点问题，政府系统作风建设持续深化。我们坚持反腐倡廉，强化党性党风党纪教育，加强重点领域、重点岗位和关键环节的管理监督，坚决查办了一批违纪违法案件，政风行风有了明显好转，政府自身建设不断加强。

　　过去的一年，新余经济社会发展砥砺前行，在巨大的压力面前能取得这样的成绩，实属不易。这得益于中央、省委省政府和市委的坚强领导，得益于市人大的法律监督和市政协的民主监督，得益于社会各界和全市广大人民群众的积极作为。在此，我代表市人民政府，向全市广大工人、农民、知识分子、干部和历任老领导、老同志，向各民主党派、工商联、无党派和社会各界人士，向驻市人民解放军、武警官兵和公安干警，向驻市中央和省属单位，致以崇高的敬意！向所有关心、支持新余发展的同志们、朋友们，表示衷心的感谢！

　　回顾过去一年的工作，我们清醒地看到，全市经济社会发展仍面临不少困难和问题：产业结构不够优，第三产业发展相对滞后，新兴产业总量偏小，转型升级任务艰巨；政府债务较重，新的税收增长点不多，财政收支平衡压力较大；生态环境、城市功能有待提升，教育、医疗、养老等公共服务水平与群众需求还有不小差距；少数干部改革创新意识不强、服务发展水平不高、破解难题能力不足等问题还不同程度存在，优化发展环境任重道远。对此，我们一定认真对待，切实加以解决。

　　二、2017年工作安排

　　2017年，是党的十九大召开之年，也是我们全面落实市第八次党代会决策部署的第一年。政府工作的总体要求是：全面贯彻党的十八大及十八大以来历次全会和中央、省经济工作会议精神，以习近平总书记系列重要讲话精神和治国理政新理念新思想新战略为指导，坚持稳中求进工作总基调，适应把握引领经济发展新常态，牢固树立和贯彻落实新发展理念，坚持以提高发展质量和效益为中心，以推进供给侧结构性改革为主线，立足“工小美”，全面做好稳增长、促改革、调结构、惠民生、防风险各项工作，促进经济平稳健康发展和社会和谐稳定。

　　全市经济社会发展的主要预期目标是：生产总值增长8.5%，财政总收入增长5%，规模以上工业增加值和主营业务收入均增长9%，固定资产投资增长12%，社会消费品零售总额增长10%，外贸出口总额增长1%，实际利用外资增长5%、省外2000万元以上项目资金增长10%，城镇居民和农村居民人均可支配收入分别增长8%和9%。居民消费价格指数控制在3%以内，完成省下达的节能减排计划任务。

　　围绕上述目标，重点做好以下工作：

　　(一)突出做大总量与优化结构并重，着力提升工业经济整体竞争力。坚持以组织申报中国制造2025试点示范城市为契机，大力发展新制造经济，调优工业结构，做大工业总量，努力推进工业经济由“新余制造”向“新余智造”转变，着力打造全省产业升级的先行区。

　　强化主导产业带动。以实施重大项目、打造龙头企业、培育产业集群为抓手，大力推动钢铁、新能源、光电信息、装备制造等四大支柱产业集约化、规模化、高端化发展。全力实施锂电、手机触控、LED和装备制造产业集群三年倍增计划，依托赣锋锂业、江西雅保、佳沃新能源等企业发展动力锂电池等系列产品，构建完整的锂电池产业链；依托沃格光电、大米智联等企业，推动手机触控产业加快形成“整机＋关键元器件”双向发展格局；依托木林森、好英王等企业，着力主攻器件封装、应用产品、配套材料三大方向，推动LED产业做大做强；依托瀚德科技、新联泰等企业，培育壮大汽车零部件、农业机械等装备制造产业，力争锂电、手机触控、LED和装备制造产业主营业务收入分别达到150亿元、140亿元、120亿元和150亿元。支持新钢公司积极发展下游产业，提升新钢本地化、区域化协作水平，实施1至2个与本地企业合作项目，力争非钢产业比重达到35%。加快推动赛维公司破产重整，大力支持企业重整后发展壮大；加大光伏电站建设力度，积极申报国家光伏“领跑者”计划。全面落实产业集群公共服务平台建设三年行动计划，强力推进电镀产业园、热处理中心等12个公共服务平台建设，确保年内建成10个。大力推进园区基础设施建设，新建标准厂房50万平方米。

　　强化企业帮扶推动。完善企业精准帮扶工作机制，着力解决制约企业发展的深层次矛盾。打好“众创业、个转企、小升规、规转股、股上市、育龙头、聚集群”组合拳，把新余打造成创业创新的洼地。提高产业引导专项资金规模和使用效率，鼓励县区设立产业引导专项资金。扩大还贷周转金规模，加大融资担保服务力度，提升担保机构担保能力。继续发挥信贷通作用，多层次、多方式支持实体经济发展。搭建全市物流信息服务平台，切实降低企业运输成本。完善与产业发展相协调的职业院校学科建设和人才培养机制，促进更多毕业生留余就业。确保新增入规工业企业30户以上。

　　强化科技创新驱动。编制制造业转型升级十年规划，推动工业和信息化深度融合。建立1家覆盖全市的科技协同创新综合服务机构，建设一批企业数字化图书馆，着力提升科技服务水平。支持企业加大科技研发投入，力争新增国家高新技术企业10家，申报省级以上科技项目40项以上，实施市级科技重点项目20项以上，新增市级科技研发平台或团队5家。全面落实国家关于依法保护产权的意见，力争每万人发明专利拥有量达2件，专利申请量达1700件，授权量达1000件。创新人才引进机制，加快培养战略性新兴产业急需人才。

　　(二)突出发展提速与质量提升并重，全力推进服务业转型升级。坚持生产性服务业与制造业融合发展、生活性服务业与扩大居民消费相互促进，全面实施服务业升级行动，大力发展新服务经济，切实提高第三产业占生产总值的比重。

　　推动传统服务业转型发展。加快推进铜锣湾商业文化广场、城北商业中心等项目建设，提升商贸服务业层次和水平。加快完善市场体系，推进佰联万家仓储物流配送中心、菜小二农产品供应链管理等项目建设。以全国性股份制商业银行为重点，全力引进金融机构或类金融机构入驻，积极发展融资租赁、商业保理、股权投资、私募基金等新业态。加快建设陆路口岸作业区和电子口岸，积极申报公共保税仓。

　　推动现代服务业提速发展。实施重大文化产业项目带动战略，重点推进体育公园、欢乐大世界、分宜麻纺小镇等项目建设，实施促进文化消费计划，扶持发展动漫产业，加快形成初具规模、富有特色的文化产业体系。推进仙女湖与上海复星集团、江西旅游集团的全方位合作，力争仙女湖景区成功创建国家5A级景区，大力推动国家全域旅游示范区创建工作。加大乡村旅游A级景区创建力度，充分挖掘红色资源、古色资源、绿色资源，打造“红、古、绿”乡村三色旅游新引擎。以国际麻纺博览会、南方农业（工程）机械展销会等会展项目为重点，大力发展会展经济。

　　推动新兴服务业蓬勃发展。集中力量和资源，大力开发新技术、创造新模式、催生新业态，推动新一代信息技术、智能制造、电子商务等为代表的新经济加快发展。重点发展工业设计、中介服务、第三方物流、节能环保服务、检验检测认证等新业态，引领制造业向价值链高端攀升；推进“互联网＋”服务业发展，促进云计算、大数据等新兴信息技术深度融入教育、文化、医疗等领域，创新商业模式，拓宽发展空间；围绕住宿餐饮、家政服务、交通出行等发展生活性分享经济，突出产能分享、科研分享、数据分享等发展生产性分享经济。

　　(三)突出招大引强与项目建设并重，加快打造经济发展新引擎。坚持以项目为总抓手，想方设法把项目引进来，千方百计把项目建起来，推动更多大项目、好项目扎根新余、开花结果。

　　狠抓重点项目建设。按照市级重点项目个数和年度投资总额不低于2016年的要求，初步安排第一批重点项目157个，年度计划投资174.8亿元。完善重点项目考核办法，推行重点项目市直主管部门、落户县区双责任制。优化项目结构，全力推进木林森LED照明、江西雅保4万吨氢氧化锂、赣锋锂业1.5万吨电池级碳酸锂、锦绣物流装备生产基地等项目建设，确保工业项目总数和投资总额不低于全市重点项目的40%。切实抓好“两厂一路”等重大基础设施项目建设，争取蒙华公司新余综合维修基地建成投运，推动新余煤储基地、铁路综合物流园等6个中心项目尽早落地，启动国电投分宜电厂铁路专用线建设，完成大唐新余二期扩建“五通一平”等工作。开展新余至吉安城际铁路项目前期工作。全力以赴推进国省道一级公路绕城项目和主城区备用水源项目开工建设，努力让市民尽快告别货车进城扰民的烦恼，加快解决市民安全饮水的后顾之忧。

　　提升招商引资实效。实施招大引强攻坚计划，紧盯产业龙头和关联企业，分门别类制定产业招商政策，力争引进国内五百强或总投资10亿元以上项目7个以上。坚持领导带头招商，加强区域驻点招商、产业招商、商（协）会招商，积极探索股权招商新模式，鼓励龙头企业进行产业链、产业群自主招商。完善返乡创业激励政策，积极推动余商回归，支持本土企业发展壮大。搭建招商信息共享平台，严格兑现招商优惠政策，建立“全流程”项目服务机制，强化从项目洽谈到建成投产各环节相互衔接。完善招商奖励机制，强化招商引资考核督查，促进项目早签约、早建设、早投产。

　　拓宽筹资融资渠道。大力推广政府和社会资本合作模式，放宽民间投资在竞争性领域的市场准入。着力构建新型政银企关系，引导银行加快信贷精准投放。探索利用政策性银行融资合作，进一步降低地方和企业融资成本。鼓励企业发行债券，加快推动企业上市融资，引导扶持更多企业开展股改、上市辅导等工作，力争3家以上企业在“新三板”挂牌。全力做好项目储备和前期工作，力争争取上级资金增长10%。

　　(四)突出基础建设与功能完善并重，切实改善城乡发展质量。坚持以人为本，加快转变城乡发展方式，完善基础设施建设，提升公共服务水平，推动城市农村“比翼齐飞”。

　　不断提升城市品位。大力实施城市升级三年行动计划，推进城市建设从扩容向提质转变，加快构建“一城四区后花园”空间布局。启动编制城区水系连通专项规划、高铁新区产城融合战略规划、袁河生态新城城市设计、高铁新区城市设计等10个规划，完成海绵城市和综合管廊规划编制。着眼“补短板、惠民生、提品质”，安排基础型、生态型、功能型市本级城建项目29个、年度计划投资12.3亿元，其中竹山北路新建、胜利北路改造、和谐大道建设等新建项目20个、年度计划投资4.4亿元，新钢路东棚户区改造、新纺棚户区改造、高铁新区地下综合管廊等续建项目9个、年度计划投资7.9亿元。扎实推进城北片区11条背街小巷、14个老旧小区、4个城中村基础设施改造，全面改善城区居民生活环境。发放廉租住房租赁补贴5000户，提高棚改货币化安置率。严格落实公交优先发展战略，加大纯电动公交车更新力度，提高公共自行车管理服务水平。加快新型智慧城市建设，启动地下管线信息化建设，促进大数据、物联网、云计算等现代信息技术与城市管理深度融合，提升城市治理和服务水平。

　　推进城乡融合发展。出台指导意见，高起点规划、高标准打造、高效率推进特色小镇建设。坚持分批建设、有序推进，找准产业定位，明确产业特色，在分宜县、渝水区、仙女湖区分别重点打造2至3个产业特色、人文底蕴和生态禀赋突出的“产、城、人、文”四位一体特色小镇。坚持市场化运作和项目带动，吸引更多社会资本参与特色小镇建设。突出村庄整治、环境美化、乡风文明、和谐平安四大重点，全面开展“整洁美丽、和谐宜居”乡村建设行动，着力抓好农村党的建设、“颐养之家”建设、经济发展、健康卫生服务、建房规划整治、环境综合整治、集镇服务管理等七项工作，努力改善农村生产生活生态条件。严格执行乡镇建设规划许可制度，制定颁布农村建房标准图谱和风貌指导手册，有效管控农村建房体量风貌。完善城乡交通体系，实施重点交通项目12个、年度计划投资7.8亿元，重点抓好仙女湖高速收费站扩建、通自然村水泥路建设147公里、县乡道升级改造20.7公里、危桥改造30座等项目建设。

　　大力发展现代农业。加快转变农业发展方式，着力构建“接二连三”的农业全产业链，在一产上提升规模、质量和效益，在二产上延伸产业链、提高农产品深加工率，在三产上拓展综合服务、仓储物流、休闲观光等功能，着力打造全国有影响的农业可持续发展示范市。增加绿色优质农产品供给，大力发展中药材、草食畜禽、旅游休闲农业等特色产业。加快推进绿色生态农业“十大行动”，推广循环农业模式，建成南英垦殖场规模化沼气发电项目。积极引导农村土地规范有序流转，推动发展多种形式的适度规模经营。

　　(五)突出污染防治与生态保护并重，大力培育绿色发展新动能。坚持在发展中保护、在保护中发展，切实加大污染源头防治力度，着力构建绿色产业体系和空间格局，争做美丽中国“江西样板”的排头兵。

　　强化环境污染防治。严格落实项目环保准入制度，从源头上防止环境污染和生态破坏。抓好重点企业废水、废气排放在线监测系统及联网建设，督促企业污染防治设施正常稳定运行。严肃查处环境违法行为，打好环境执法组合拳，实现环境违法行为零容忍，促进环境守法成为常态。

　　持续推进“三净”行动。全面推进“河长制”，开展仙女湖湖泊保护治理、袁河新余段水环境综合整治，加快农村生活污水处理设施建设，确保主要河流交接断面和仙女湖、孔目江两个饮用水源地水质达标率均达100%，确保全国水生态文明城市建设试点顺利通过验收。系统推进大气污染物联合治理，重点加强工业点源污染治理、城市扬尘污染防治、机动车尾气污染控制，持续改善城区空气质量。以农用地和重点行业企业用地为重点，创新重金属土地污染治理模式。切实做好“五山”保护，呵护渝钤大地美好家园，让新余的天更蓝、水更清、山更绿，空气更清新。

　　建立健全生态文明制度。按照“源头严防、过程严管、后果严惩”的思路，加快建立系统完整的生态文明制度体系。严格落实环境保护责任制和耕地保护责任制，将生态文明建设工作纳入领导干部政绩考核体系。开展自然资源统一确权登记，编制自然资源资产负债表，稳步推进领导干部自然资源资产离任审计，努力守护美丽新余的生态屏障。

　　(六)突出守住底线与补齐短板并重，努力扩大人民群众获得感。坚持以人民为中心，全力保障群众基本生活，努力办好45件民生实事，不断提升基本公共服务水平，让改革发展成果惠及全市人民。

　　提升民生保障水平。完善“双创”服务平台，加大创业担保贷款发放力度，全面推进大众创业万众创新。提高五险同步参保覆盖面，加快城乡居民医保整合步伐，推进大病医保精准扶贫，适度提高未参保城镇大集体企业退休人员等养老生活补助标准。推进民办养老机构达标、社区居家养老示范点建设，创新农村养老模式，努力实现全市行政村“颐养之家”覆盖率超80%。提高社会救助工作水平，确保城乡低保应保尽保。抓好以光伏发电为重点的产业扶贫，力争有条件的行政村各建一座50千瓦的光伏扶贫电站，增加村级集体收入，确保现行标准下农村贫困人口全部脱贫。

　　繁荣各项社会事业。坚持教育优先，深化“小学对口直升初中”“多校划片”招生模式改革。加快公办幼儿园和城区学校网点建设，支持民办幼儿园发展，推进市城北幼儿园、市九中等7所学校教学楼建设，开工建设埠上小学等3所学校，谋划市老年大学二校区改造。扩大现代学徒制试点范围，推进产教融合、校企合作。持续深化公立医院综合改革，加快推进卫生应急服务能力建设，适时启动市中医院迁建及市妇幼保健院改扩建项目。进一步提高公共文化服务能力和水平，推动传统文化进社区，在全省率先实现公共文化标准化、均等化，努力争创全国文化消费试点城市。唱响“新余有爱”志愿服务品牌，加快推进全国文明城市创建活动，着力提升市民文明素质。新建一批社区、行政村健身路径，大力发展竞技体育，促进全民健身与健康新余深度融合。关心支持妇儿、青少年、老龄事业，发展慈善和残疾人事业。加强国防后备力量和人民防空建设，推进“双拥”和优抚安置。做好新形势下的科普、气象、史志、档案、外事侨务、民族宗教、驻外机构、防灾减灾、邮政通讯等工作。

　　创新社会治理方式。注重联动融合、开放共治，积极推进社会治理社会化、法治化、智能化、专业化。严厉打击各类违法犯罪行为，大力实施智慧天网完善工程，加快实现集镇交通信号灯全覆盖，着力构建城乡立体化、信息化社会治安防控体系，切实增强人民群众安全感。深入开展法治新余建设，健全覆盖城乡的法律服务体系，完善人民调解、行政调解、司法调解联动工作机制，畅通信访渠道，积极化解社会矛盾。牢固树立安全发展理念，全面落实安全生产责任制，确保安全生产形势稳定。实施“食安前哨”工程，推进国家食品安全城市创建试点建设，提升食品安全综合治理水平。完善应急体系，加强应急联动，提高对各类突发事件的预警预防和应急处置能力。

　　(七)突出深化改革与服务发展并重，不断增强经济社会发展内生动力。坚持以改革倒逼政府职能转变，以改革实现供求关系新的动态平衡，以改革营造一流投资环境，不断激发经济社会发展新活力。

　　持续推进“放管服”改革。不断深化行政审批制度改革，做好国务院、省政府取消和下放行政审批事项的衔接工作。按照“宽进严管”原则，加快推进事前审批向事中事后监管转变。全面推行网上“一站式”审批制和行政审批事项目录管理制，规范行政审批行为。推进机构编制管理改革，进一步整合内设机构。按照精干高效原则，深入推进园区内部管理体制改革。着力推进“互联网+政务服务”，实现办事服务“一号申请、一窗受理、一网通办”。抓好公共资源交易平台网上交易系统与上级电子服务系统的对接，加快形成互联互通。积极策应赣西（新余）跨行政区转型合作示范区建设，启动一批实质性合作项目。

　　深入推进供给侧结构性改革。加快财税体制改革，健全全口径预算制度，完善转移支付资金分配机制和一般性转移增长机制。继续清理各项行政事业性收费、政府性基金，降低企业制度性成本。注重运用市场机制，切实化解过剩产能，妥善处置“僵尸企业”。分类推进国有企业改革，实现国有资产保值增值。积极发展住房租赁市场，鼓励房地产开发企业调整营销策略，有效化解房地产库存。稳妥推进出租车行业改革。加强金融风险化解处置，强化政府债务管理，有序降低实体经济杠杆水平，维护地方金融稳定。

　　加快推进社会管理体制改革。改革企业投资核准制和备案制，最大限度地缩小企业投资核准范围，落实企业投资自主权。加快培育发展各类中介组织，加大政府购买服务力度。全面推进城市管理和执法体制改革，切实理顺城市管理体制，提高城市管理效率。建立城乡统一的建设用地市场，完善土地增值收益分配机制，促进存量低效用地的流转和再开发。积极推进农村集体经济组织产权股份合作制度改革试点，探索农村土地承包经营权等产权抵押融资模式。加快推进社区数字化综合服务平台与部门平台深度融合，系统推进社区标准化、信息化建设，完善网格化管理、社会化服务。

　　各位代表！全面完成各项目标任务，对政府工作提出了新的更高要求。我们将坚持问题导向、市场导向、需求导向，切实加强政府自身建设，努力打造人民满意政府。

　　着力推进依法行政。认真落实法治政府建设实施纲要，健全立法机制，提高政府立法质量。严格执行“三重一大”报告制度，强化政府重大行政决策的合法性审查，提高依法科学民主决策水平。全面落实行政执法责任制，切实规范行政执法行为。加快推进综合执法改革试点工作，实现行政处罚与刑事司法无缝对接。深入推进政务公开，完善新闻发言人制度，提高政府运作透明度。自觉接受人大法律监督、政协民主监督和社会各界监督，高质量办理人大议案、政协提案及其意见建议。

　　切实加强廉政建设。严格执行党风廉政建设各项规定，全面落实主体责任和一岗双责，旗帜鲜明地反对腐败，始终把纪律挺在最前面。切实加强工程建设、房地产开发、土地管理、矿产资源开发等重点领域和关键环节的规范管理，严肃查处发生在群众身边的不正之风和腐败问题。建立完善容错纠错机制，旗帜鲜明地为改革者鼓劲、为实干者撑腰、为担当者担当、为负责者负责。

　　持续改进工作作风。完善干部考评体系和奖惩机制，健全部门间协调配合机制，加大督查督办力度，着力治理庸懒散，从严整治不作为、乱作为、慢作为，切实增强行政执行力。认真落实首问责任制、限时办结制、服务承诺制等制度，切实提高工作效能。坚持从群众中来，到群众中去，深入开展调查研究，掌握实情、解决问题、推动工作。持续改进行政管理和服务方式，不断优化政务环境，努力提升政府公共服务满意度。

　　各位代表！小城崛起风正劲，扬帆起航恰当时。让我们紧密团结在以习近平同志为核心的党中央周围，在省委、省政府和市委的坚强领导下，凝心聚力，开拓进取，真抓实干，奋发有为，为加快建设“三区两典范”、再铸新余“工小美”辉煌而努力奋斗！
